# Structural Characterizations of Palladium Clusters Prepared by Polyol Reduction of [PdCl_**4**_]^**2**−^ Ions

**DOI:** 10.1155/2016/9073594

**Published:** 2016-03-17

**Authors:** Loredana Schiavo, Lucrezia Aversa, Roberta Tatti, Roberto Verucchi, Gianfranco Carotenuto

**Affiliations:** ^1^Physics Department, University of Trento, Via Sommarive 14, 38123 Povo, Italy; ^2^Institute for Polymers, Composites and Biomaterials (IPCB), National Research Council (CNR), Piazzale E. Fermi 1, 80055 Portici, Italy; ^3^Institute of Materials for Electronics and Magnetism (IMEM), National Research Council (CNR), Via alla Cascata 56/C, 38123 Povo, Italy

## Abstract

Palladium nanoparticles are of great interest in many industrial fields, ranging from catalysis and hydrogen technology to microelectronics, thanks to their unique physical and chemical properties. In this work, palladium clusters have been prepared by reduction of [PdCl_4_]^2−^ ions with ethylene glycol, in the presence of poly(N-vinyl-2-pyrrolidone) (PVP) as stabilizer. The stabilizer performs the important role of nucleating agent for the Pd atoms with a fast phase separation, since palladium atoms coordinated to the polymer side-groups are forced at short distances during nucleation. Quasispherical palladium clusters with a diameter of ca. 2.6 nm were obtained by reaction in air at 90°C for 2 hours. An extensive materials characterization by transmission electron microscopy (TEM), X-ray diffraction (XRD), X-ray photoelectron spectroscopy (XPS), and other characterizations (TGA, SEM, EDS-SEM, and UV-Vis) has been performed in order to evaluate the structure and oxidation state of nanopalladium.

## 1. Introduction

Palladium nanoparticles can be exploited for technological applications in different industrial fields (e.g., catalysts, electrically conductive inks, materials for hydrogen storage, and sensors) [1–4]. Their physicochemical properties depend strictly on their size; thus chemical methods aimed at achieving tailored nanoparticles are strictly required.

Many studies on the preparation of palladium particles, nanoparticles, and clusters (i.e., nanoparticles with a size of few nanometers) are available in the literature [5–16]. Under uncontrolled conditions “palladium black,” that is, agglomerated fine palladium particles (average size less than 0.1 *μ*m), is produced by using the “polyol process” approach. This method involves the chemical reduction of a metal salt by refluxing it in a liquid polyol, for example, ethylene glycol, glycerol, diethylene glycol, and triethylene glycol, which acts as solvent for the precursor and as dispersing medium for the resulting nanoparticles [5–7]. Palladium particles in the submicrometer range have been obtained by using palladium(II) tetramine complex as precursor and an auxiliary reducing agent (hydrazine hydrate); this reaction was performed at low temperature (below 0°C) [6]. This process was improved by changing the type of polyol; thus submicrometer-sized monodisperse quasispherical palladium particles with an average diameter of ca. 0.15 *μ*m were obtained [7]. However, it has been noted that the use of stabilizers makes possible obtaining shape-controlled palladium particles on a nanoscopic scale [8–11]. The chemical synthesis of palladium nanoparticles with different stabilizers as, for example, organic ligands, salt/surfactants, polymers, and dendrimers, allows obtaining monodispersed nanosized palladium with well-controlled particle size and shape in the 1–100 nm range *‎*[13]. Poly-(N-vinyl-2-pyrrolidone) (PVP) is one of the most investigated polymers used to stabilize metal nanoparticles [11–16] due to the presence of a chelating agent that readily leads to very small particles. The morphology of the palladium nanoparticles obtained in presence of PVP also depends on the reaction conditions like temperature, reaction time, metallic precursor type, and so forth *‎*[11], so it can be finely tuned.

Here, palladium clusters have been prepared by a very simple synthesis technique based on the polyol reduction of K_2_PdCl_4_ in ethylene glycol and in air at mild temperature conditions, using (PVP) as polymeric surface stabilizer. The reaction mechanism involved in the palladium ions reduction is the following:

(1) $$\begin{array}{c} \text{OH–C}{\text{H}}_{2}\text{–C}{\text{H}}_{2}\text{–OH}+\text{P}{\text{d}}^{2+}\overset{-2{\text{H}}^{+}}{\to }\text{Pd}\left(\;0\;\right)+2\text{H–CHO} \end{array}$$

In this work, polymer-embedded palladium clusters of quasispherical shape have been obtained. The experimental conditions (in particular the 90°C temperature but also the type/amount of salt, the concentrations, etc.) have been selected to reduce completely the precursor and get extremely small clusters. The structure and the surface oxidation state of palladium clusters were extensively studied in order to evaluate the effects of a noninert atmosphere on their synthesis. In particular, the morphology was characterized by transmission electron microscopy (TEM) and the crystalline nature studied by X-ray diffraction (XRD). Further characterization has been done by X-ray photoelectron spectroscopy (XPS), thermogravimetric analysis (TGA), energy-dispersive X-ray spectroscopy (EDS-SEM), and optical spectroscopy (UV).

## 2. Experimental

Poly(N-vinyl-2-pyrrolidone) (Aldrich, *M* = 10,000 gmol^−1^) was dissolved in ethylene glycol (Aldrich, 99.0%) and the resulting solution was placed in a thermostatic bath at 90°C for 120 minutes in air. Separately, a small volume of a concentrated K_2_PdCl_4_ (Aldrich, 99.99%) solution in ethylene glycol was prepared and it was quickly added to the PVP/EG solution at ambient atmosphere under vigorous stirring. The PVP concentration in ethylene glycol was 30 mM and the molar ratio Pd(II) :  PVP (the repeating unit) was 1 : 10 *‎*[7]. The solution color changed from light-brown to black when the [PdCl_4_]^2−^ ions disappeared and transformed into palladium clusters *‎*[14]. After a thermal annealing of 2 h the solution was cast into a large amount of pure acetone (Aldrich) and the system was left at room temperature for 12 h. Subsequently the acetone supernatant was removed and replaced with fresh acetone (100 mL), and the system was treated in bath sonication for 30 minutes. This washing procedure was repeated three times. The PVP completely flocculated from this nonsolvent liquid (ethylene glycol/acetone mixture), coprecipitating the coordinated palladium clusters.

The morphology of PVP-embedded palladium clusters was studied by transmission electron microscopy (TEM) using a FEI Quanta 200 FEG operated at 200 kV. The observations were carried out using Formvar-coated grid. In particular, the Pd-PVP system was dissolved in ethanol at room temperature and a drop of this solution was placed on the Formvar-coated copper grid. X-ray powder diffraction (XRD) patterns were recorded on a Bruker D8 advance X-ray diffractometer employing CuK*α* radiation (*λ* = 1.5406 Å) with 40 kV and 40 mA. The PVP nanocomposites were evaluated by thermogravimetric analysis by heating the sample under fluxing air at a rate of 10°C/min (TGA Q5000, TA Instruments). The energy-dispersive X-ray spectroscopy (EDS-SEM) measurements were obtained by using a Scanning Electron Microscopy FEI QUANTA 200 FEG. UV-Vis absorption spectra (UV) were measured on Lambda 850 (PerkinElmer) spectrophotometer. X-ray photoelectron spectroscopy (XPS) has been carried out in a UHV apparatus equipped with an X-ray source (Mg *K*_*α*_ photon at 1253.6 eV), while photoelectrons are analyzed through a VSW HA100 hemispherical analyzer with a total energy resolution of 0.86 eV. Core level binding energies (BE) have been normalized using as reference the Au 4f_7/2_ core level signal at 84 eV, acquired from a sputtered gold foil. The photoemission core levels (C1s, N1s, O1s, and Pd3d) have been analyzed through Voight line shape deconvolution after background subtraction of a Shirley function. The typical error for peak position is ±0.05 eV while for the area evaluation it is about ±2.5%. In order to perform XPS analysis, the sample powders were dispersed in isopropyl alcohol, dropped on a Si(100) substrate previously cleaned in a sonication bath of trichloroethylene-acetone-isopropyl alcohol, and left to dry in air for 24 hours, before being inserted in the UHV chamber.

## 3. Results and Discussion

**Figure 1:**
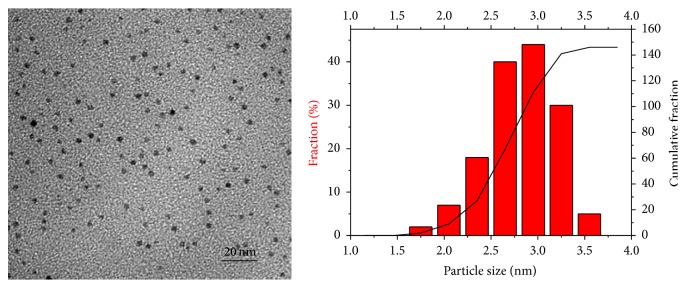
TEM micrographs of palladium clusters embedded in PVP produced by reduction of [PdCl_4_]^2−^ at 90°C and palladium particle size distribution (average diameter = 2.6 ± 0.3 nm).

Figure 1 shows the TEM micrographs of the smallest achieved Pd nanoparticles. Very small palladium crystals with an average size of ca. 3 nm resulted from reducing the [PdCl_4_]^2−^ ions at 90°C for 120 minutes. They are monodispersed clusters characterized by a quasispherical shape [14, 15].

**Figure 2:**
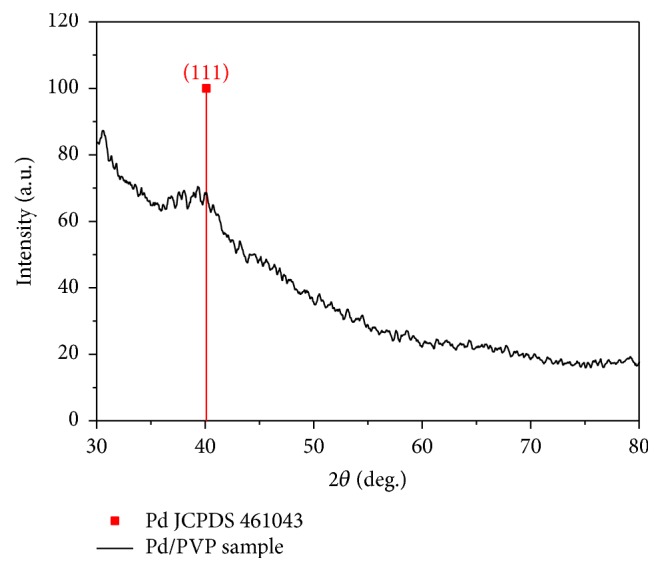
XRD of palladium clusters produced by reduction of [PdCl_4_]^2−^ at 90°C.

The XRD pattern of palladium clusters obtained in the same experimental conditions is shown in Figure 2. A broad peak located at 2*θ* = 40.1° corresponding to the (111) lattice plane of palladium diffraction pattern, according to JCPDS-46104, was observed *‎*[14]. The peak was broad and low in intensity owing to the extremely small crystal size. The mean particle size was estimated by the Scherrer equation applied on (111) diffraction peak *‎*[17], and it was ca. 5 nm which slightly overestimates the real size obtained by TEM.

**Figure 3:**
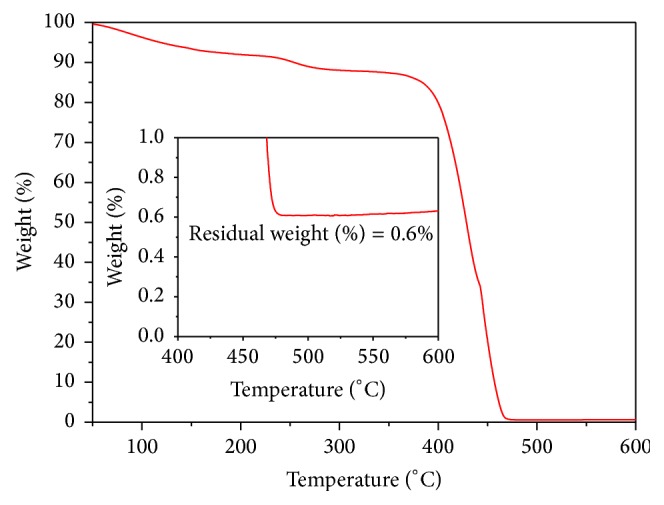
TGA in air of palladium clusters embedded in PVP produced by reduction of [PdCl_4_]^2−^ at 90°C.

The thermogram of a palladium/PVP sample is shown in Figure 3. Most of the weight loss is in the range of 400°C–460°C and the residual weight is of 0.6% (see inset). The amount of residual weight that corresponds to the synthetized Pd(0) phase is higher than the expected theoretical value (0.1 wt.%), probably due to the PVP loss during the washing cycles and to traces of impurities coming from K_2_PdCl_4_.

**Figure 4:**
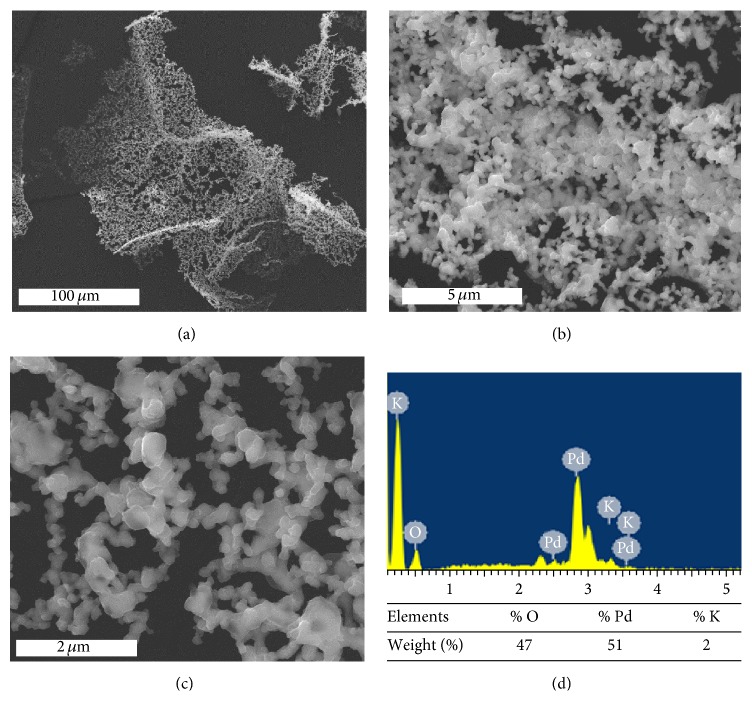
SEM micrographs of the residual palladium clusters embedded in PVP obtained by TGA tests in air ((a), (b), and (c)) and EDS composition percentages (d) of residual material obtained from TGA test.

Energy-dispersive X-ray spectroscopy (EDS-SEM) was used to determine the chemical composition of TGA test residual material (Figure 4(d)). A high spongy palladium structure is well visible in the SEM micrographs (Figures 4(a), 4(b), and 4(c)) of this sample and its chemical composition is mainly composed by oxygen and palladium together with traces of other elements, coming from the reagents.

**Figure 5:**
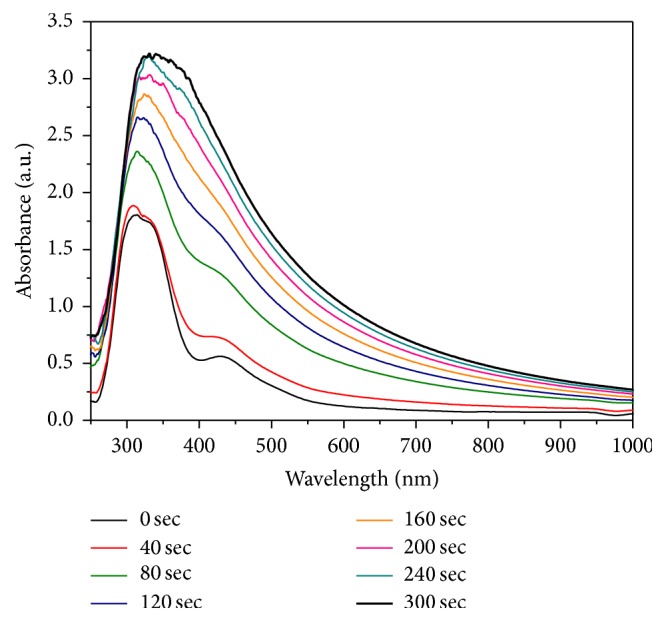
UV-Vis absorption spectra at different reaction times during the reduction of [PdCl_4_]^2−^ at 90°C.

Figure 5 shows the temporal evolution of UV-Vis absorption spectra of the reactive system. The freshly prepared solution was promptly placed in the UV-Vis spectrophotometer, previously thermostated at 90°C, to evaluate the variation of the absorption peaks during the reaction. As shown in Figure 5, at reaction beginning (0–20 s curves), two strong characteristic absorption peaks at ca. 305 nm and 330 nm are present, corresponding to [PdCl_4_]^2−^ ion [8, 15]. In addition, another peak at ca. 440 nm that should correspond to [PdCl_4_]^2−^ ion was observed, but it is too weak to be clearly identified. It can be seen that no significant change for the absorption peaks was observed after 20 s. When the reaction proceeded for 100 s, the peak at 330 nm disappeared almost completely and the absorption spectrum exhibited a typical light-scattering phenomenon, thus indicating the formation of palladium nanoparticles. The light-scattering is further enhanced with time, due to the increase of Pd nanoparticles, reaching an intensity maximum after 140 s. Prolonged heating did not give any variation for the absorption spectrum. During the process the solution color changed from light-yellow to black because of the [PdCl_4_]^2−^ ions disappearance and palladium clusters formation, indicating that the reduction process was complete after 140 s.

**Figure 6:**
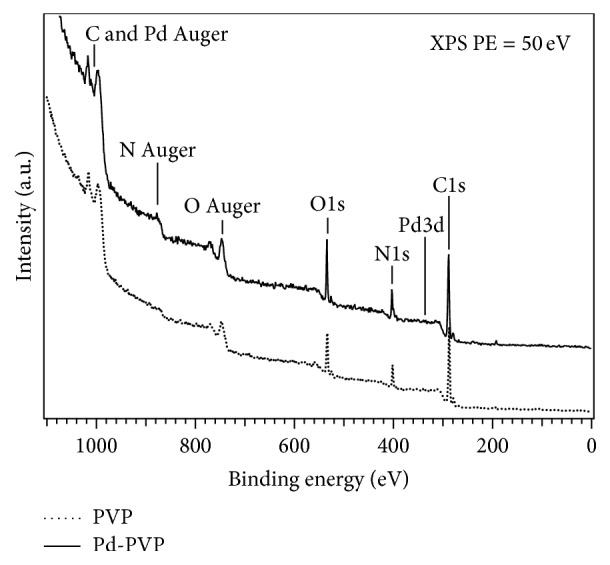
XPS wide-range spectra of Pd nanoparticles in PVP and of a PVP untreated sample for reference.

XPS was employed to investigate the chemical state of the palladium nanoparticles and qualify the interaction between PVP molecules and palladium atoms during the formation of PVP-capped palladium nanoparticles.  Figure 6 shows the wide binding energy (BE) range spectrum for the PVP-capped Pd nanoparticles compared to the PVP reference spectrum, putting in evidence the presence of all the expected chemical species, that is, C, O, and N (both Auger emission and core levels are clearly identifiable), and a very weak contribution from Pd3d core level.

**Figure 7:**
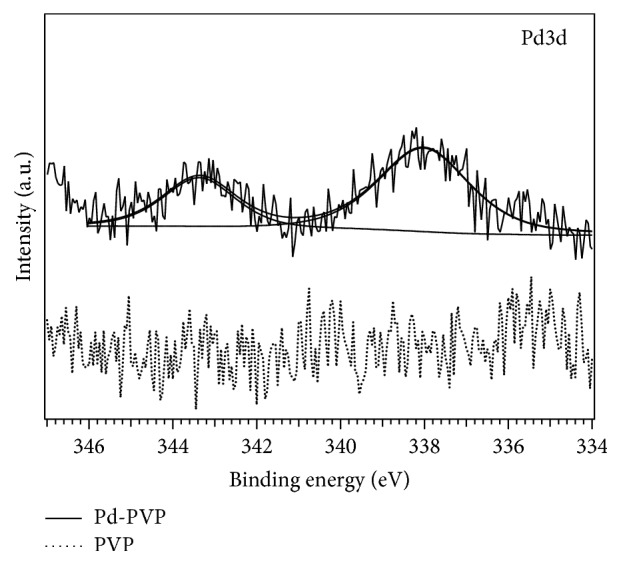
XPS Pd 3D spectra of Pd nanoparticles in PVP and of a pure PVP sample for reference.

All core levels are shifted towards higher BE, a phenomenon that is related to charging effects during the analysis due to the presence of the polymeric nonconducting material *‎*[18]. Such a shift can be evaluated to be about +2.5 eV from the main C1s component that should be at 285 eV.  Figure 7 shows the Pd3d region for a PVP film and PVP-capped palladium nanoparticles. Pd3d core level is located at 338.0 eV, with a spin-orbit splitting of 5.27 eV. Taking into account charging effects, the peak position for Pd3d can be rescaled to lower BE suggesting that the as-prepared Pd nanoparticles are in a metallic chemical state rather than oxidized [19–21]. The Pd core level intensity is very low, as it is shown with the comparison of a PVP sample without Pd particles (Figure 7). Such a low intensity can be probably related also to the presence of the capping PVP layer, leading to a strong photoemission signal decrease as the escape depth of Pd photoelectrons is in the range of few nanometers *‎*[20]. The surface atomic percentage of each chemical species has been evaluated from core level intensity and for Pd is 0.1%, a value close to the XPS sensitivity but in agreement both with the low density of the nanoparticles observed by the TEM images and with energy-dispersive X-ray spectroscopy (EDS-SEM) (Table 1). Considering other chemical species, the following percentages have been found: 76.1% for C1s, 12.4% for O1s, and 11.4% for N1s, in good agreement with the chemical formula of PVP showing theoretical atomic percentages of 75.0% for C, 12.5% for O, and 12.5% for N.

The comparison of the weight percentages of palladium included in the residual of TGA in air (Figure 4(d)) with the weight percentages of palladium embedded in PVP (Table 1) shows that the palladium clusters embedded in PVP are very diluted in the protective polymer.

## 4. Conclusions

Extremely small and monodispersed palladium clusters (average diameter 2.6 nm by TEM) have been produced by reduction of [PdCl_4_]^2−^ ions with ethylene at 90°C without inert atmosphere and in the presence of PVP as surface stabilizer. These PVP-embedded palladium clusters can have several different technological applications ranging from catalysis to membranes for hydrogen-storage, and so forth. We have presented a complete characterization of Pd clusters with different ex situ techniques, aimed at studying their properties from different points of view, from structural/morphological characteristics up to chemical composition and Pd valence state. The selected reaction conditions like temperature, reaction time, metallic precursor type, and so forth play an important role in controlling both the size of synthesized palladium nanoparticles and their chemical properties and smallest and nonoxidized particles have been obtained, making them suitable candidates for the mentioned applications.

## Figures and Tables

**Table 1:** Weight and atomic percentages obtained by EDS and XPS analysis for palladium clusters embedded in PVP produced by reduction of [PdCl_4_]^2−^ at 90°C.

Elements	Weight (%) from EDS	Atomic (%) from EDS	Atomic (%) from XPS
% C	76.7	82.0	76.1
% O	22.1	17.8	12.4
% Pd	1.2	0.1	0.1
